# circ*SPG21* protects against intervertebral disc disease by targeting miR-1197/*ATP1B3*

**DOI:** 10.1038/s12276-021-00674-z

**Published:** 2021-10-06

**Authors:** Yizhen Huang, Zhenlei Zhang, Jianle Wang, Shuying Shen, Teng Yao, Yining Xu, Zizheng Chen, Bin Fang, Jianjun Ma

**Affiliations:** 1 Department of Orthopaedic Surgery, Sir Run Run Shaw Hospital, Zhejiang University School of Medicine, Zhejiang, China; 2 Key Laboratory of Musculoskeletal System Degeneration and Regeneration Translational Research of Zhejiang Province, Zhejiang, China; 3 Shaoxing University School of Medicine, Shaoxing, China; 4 Department of Spine Surgery, Shaoxing Central Hospital, China Medical University, Shaoxing, China

**Keywords:** Mechanisms of disease, Long non-coding RNAs

## Abstract

The abnormal expression of circular RNAs (circRNAs) is associated with numerous human diseases. This study investigated the mechanism by which circRNA acts as competitive endogenous RNA in the regulation of degenerative intervertebral disc disease (IVDD). Decreased expression of circSPG21 was detected in degenerated nucleus pulposus cells (NPCs), the function of circSPG21 in NPCs was explored and verified, and the downstream target of circSPG21 was investigated. The interaction between circSPG21 and miR-1197 and its target gene (*ATP1B3*) was studied by online database prediction and molecular biological verification. Finally, the circSPG21/miR-1197/ATP1B3 axis was verified in the mouse tail-looping model. The expression of circSPG21 in the nucleus pulposus in IVDD was directly related to an imbalance of anabolic and catabolic factors, which affected cell senescence. circSPG21 was found to play a role in human NPCs by acting as a sponge of miR-1197 and thereby affecting *ATP1B3*. The regulation of circSPG21 provides a potentially effective therapeutic strategy for IVDD.

## Spinal disease: Circular RNA protects against degenerative disc disease

A type of non-coding circular RNA implicated in maintaining the cartilaginous discs found between adjacent vertebrae of the spine offers a promising new therapeutic target for treating chronic lower back pain. A team from China led by Bin Fang of China Medical University, Shaoxing, and Jianjun Ma of Zhejiang University School of Medicine, Hangzhou, found that people with degenerative intervertebral disc disease (IVDD) express low levels of a circular RNA known as circSPG21 in the cells that populate the area between spinal discs. The researchers showed how this RNA interacts with a regulatory microRNA to affect the function of a protein involved in warding off cell death. Injections of circSPG21 alleviated the symptoms of IVDD in a mouse model, highlighting its therapeutic potential in humans.

## Introduction

Degenerative disc disease is an age-related disease^1^. With increasing age, the disc gradually loses flexibility, elasticity, and shock absorption function, and the fibrous rings that surround the disc become fragile and tear easily^2^. Simultaneously, the central nucleus pulposus (NP) loses water and atrophies. Lower back pain caused by degenerative changes in the intervertebral disc is an important reason for the decline and loss of the working ability of the population, which has severe social and economic impacts. The current clinical treatment strategy for this disease primarily focuses on alleviating symptoms and cannot prevent or treat disc degeneration^3^. Therefore, an in-depth understanding of the mechanism of disc degeneration has crucial scientific value and could provide a theoretical basis for identifying new prevention and treatment strategies for disc degeneration^4^.

In contrast to traditional linear RNA (including 5′ and 3′ ends), circular RNA (circRNA) has a closed ring structure that is unaffected by RNA exonucleases, is more stably expressed, and is not easily degraded^5^. Some circRNAs have microRNA (miRNA) response elements and interact with miRNAs. Because of the high expression level and stability of circRNA, it is used as a competitive endogenous RNA (ceRNA)^6^. circRNA competes with miRNA to prevent the posttranslational inhibition of miRNA and target coding RNA, ultimately regulating the expression levels of target genes^7^.

In a study of intervertebral disc degeneration, circVMA21 was found to act as a sponge of miR-200c to regulate X-linked inhibitor-of-apoptosis protein (XIAP) and affected cell apoptosis and the imbalance of extracellular matrix anabolism and catabolism. Thus, circVMA21 could be used as a prognostic biomarker of intervertebral disc disease (IVDD) and a potential therapeutic target^8^. In a study of the functional mechanism of circRNA in the development of intervertebral disc degeneration, circERCC2 was found to alleviate IVDD by targeting the miR-182-5p/SIRT1 axis; it is therefore now used as a prognostic marker and therapeutic target for IVDD^9^.

miRNAs are noncoding single-stranded RNA molecules of ~22 nucleotides encoded by endogenous genes^10^. They promote target gene degradation or posttranscriptionally inhibit translation. miR-199a-5p promotes NP cell apoptosis and IVDD by inhibiting SIRT1-dependent p21 deacetylation, and miR-199a-5p might thus be an important therapeutic target for IVDD^11^. miR-532-3p inhibits nonsmall cell lung cancer (NSCLC) via forkhead Box P3 (*FOXP3*), suggesting that the miR-532-3p/*FOXP3* axis could be a potential therapeutic target^12^. At present, T-box transcription factor 1 (*TBX1)* is considered one of the core genes related to congenital heart disease, and miR-144 plays an inhibitory role in cardiomyocytes through the TBX1/Janus kinase 2/signal transducer and activator of transcription 1 axis^13^.

*ATP1B3* encodes ATPase Na^+^/K^+^ transporting subunit beta 3, a complete membrane protein that establishes and maintains the electrochemical gradient of Na^+^ and K^+^ ions across the plasma membrane. This protein is involved in transmembrane transport, maintaining neuromuscular excitation, and regulating osmotic pressure. As exercise changes the expression of Na^+^/K^+^ ATPase in skeletal muscle^14^ and the intervertebral disc is a load-bearing organ in the spine whereas NP tissue is rich in water, we hypothesize that Na^+^/K^+^ ATPase is associated with disc degeneration.

In this study, a circRNA derived from the autosomal recessive spastic paraplegia (*SPG21*) gene^15^ (designated circ*SPG21*; also referred to as hsa_circ_0035875 in CircBase (http://www.circbase.org)) was identified in the NP, and its role was systemically investigated in cell and animal models of IVDD. circ*SPG21* is rich in miRNA-binding sites and plays a role as a ceRNA. circ*SPG21* acts as a sponge of miR-1197, which regulates the target gene *ATP1B3*^16^. It was found that circRNA acts as ceRNA to regulate the activity and function of nucleus pulposus cells (NPCs) and the pathological process of IVDD.

## Materials and methods

### Human intervertebral disc tissue

Human NP tissues were collected, preserved, and studied according to the protocols and guidelines of the Ethics Committee of Sir Run Shaw Hospital (Zhejiang, China). Control disc tissue was removed from patients with lumbar fractures who had no history of degeneration, and pathological tissue was removed from patients with disc herniation during disc replacement. All operations and risks were reported to the patient, and informed consent was provided prior to tissue collection. Patient information is included in the Supplementary Table 1^17^.

### Mouse disc degeneration model

All mice were purchased from Shanghai SLAC Laboratory Animal Co., Ltd. (Shanghai, China). All animal experiments were performed in accordance with the principles and procedures of the National Institutes of Health Guide for the Care and Use of Laboratory Animals and the guidelines for animal treatment of Sir Run Run Shaw Hospital (Zhejiang University-affiliated, Hangzhou, Zhejiang). Six-week-old C57BL/6 mice were selected, and the tails of the mice were fixed with a thin wire after anesthesia. The tail was bent into a ring shape and held in place for one month to induce chronic degeneration of the intervertebral disc under simulated pressure^18^.

### Intraperitoneal injection of circ*SPG21*

circ*SPG21*-wt and circ*SPG21*-mut adenoviruses were constructed by Hanbio Biotechnology (Shanghai) Co. Ltd. After successful tail model generation, the mice were intraperitoneally injected with adenovirus solution once per week for 8 weeks. A 100 μL solution containing circ*SPG21-*wt or circ*SPG21*-mut was slowly injected into the abdominal cavity.

### NPC culture

NPCs were isolated from humans and mice and cultured in Dulbecco’s modified Eagle’s medium containing 10% fetal bovine serum (Thermo Fisher Scientific, Waltham, MA, USA). The cells were cultured in a humidified environment at 37 °C with 5% CO_2_ and 95% air. To ensure stability, NPCs were used within three generations.

### Bioinformatics analysis

Target microRNAs of circSPG21 were identified using two online databases (TargetScan: http://www.targetscan.org/and miRanda: http://www.microrna.org/) and RNAhybrid analysis software. Three online databases were used to predict target genes of miR-1197 (TargetScan, miRanda, and miRTarBase: http://mirtarbase.mbc.nctu.edu.tw/php/download.php). The corresponding binding sites were identified using online software (CircInteractome: https://circinteractome.nia.nih.gov/index.html and TargetScan).

### RNA extraction and RT-qPCR analysis

According to the manufacturer’s instructions, total cellular RNA was extracted from cultured NPCs using TRIzol reagent (Invitrogen, Carlsbad, CA, USA). miRNA was extracted using an miRNA extraction kit (CWBIO, Beijing, China). RNA was stored at −80 °C, and 1.0 μg of total RNA was used for reverse transcription using an miRNA cDNA kit or a HiFiScript cDNA kit (CWBIO, Beijing, China) to study miRNA and mRNA expression.

The amplification reaction contained 1 µL cDNA, 5 µL SYBR Green Master Mix (Yeasen Biotech Co., Ltd, Shanghai, China), 1 µL primer (TsingKe, Hangzhou, China), and 3 µL water, for a total of 10 µL. The mixture was denatured at 95 °C for 5 s and amplified at 60 °C for 24 s (40 cycles). An ABI 7500 Sequencing Detection System (Applied Biosystems, Foster City, CA, USA) was used to establish an amplification reaction in a 10 μL volume containing amplification primers and Supermix (including ROX; CWBIO). Please refer to the Supplementary Table 2 for all primer sequences.

### X-ray analysis

Samples were fixed in polyoxometalate and stored in 70% ethanol. The samples were scanned at 45 kV and 50 mA for 5 s, and the data were analyzed using ImageJ software (National Institutes of Health, Bethesda, MD, USA).

### Adenovirus overexpression

To construct the recombinant adenovirus expressing circ*SPG21* under the control of the mouse cytomegalovirus (CMV) promoter, PCR-cloned circular gene cDNA (Ad-circ*SPG21*) and the green fluorescent protein gene (Ad-*GFP*) were inserted into the pAdEasy-EF1-MCS-CMV-GFP vector (Han Biological, Shanghai, China). Cells were infected with the virus, and the amount of virus was adjusted according to the virus titer and the number of cells (virus amount = multiplicity of infection × cell number/virus titer (plaque-forming units/mL) × 1000). After 4 h of infection, the medium was changed, and green fluorescence was observed under a fluorescence microscope to determine whether overexpression was successful^19^.

### RNA interference and overexpression

siRNA-mediated gene knockout suppressed circ*SPG21* expression. Three different siRNAs were designed and tested. Specific inhibitors and mimics (RiboBio, Guangzhou, China) were used to inhibit or induce the expression of miR-1197. Lipofectamine RNAiMAX transfection reagent (Thermo Fisher) was used to study siRNAs, miR-1197 inhibitors, and mimetics. NPCs were seeded in six-well plates, and transfection was performed after they covered 60–80% of the area. Then, 9 μL of Lipofectamine RNAiMAX transfection reagent and 3 μL of siRNA (or inhibitors, mimetics) were diluted with 150 μL of Opti-MEM Medium each (Thermo Fisher). After the two dilutions were mixed, they were allowed to stand at room temperature for 5 min. Next, 250 μL of the mixture was added to the six-well plates for transfection. The cells were cultured at 37 °C for 48 h and then used in the experiment.

### β-galactosidase staining

NPCs were stained with a senescence β-galactosidase staining kit (C0602, Beyotime, Shanghai, China). After fixing the cells, they were stained with mixed staining solution for 8 h (37 °C). After removing excess dye, images were acquired under a microscope^20^.

### Alcian staining

Alcian staining was performed using a kit (G1563, Solarbio, Beijing, China). NPCs were added to 24-well plates and fixed with formalin. After adding the acidification solution, the cells were stained for 30 min. Excess dye was removed, and images were acquired using a scanner (V600, EPSON, Nagano, Japan)^21^.

### Western blotting

The medium was removed from the wells, and the cells were washed with phosphate-buffered saline (PBS). Human or mouse NPCs were lysed using radioimmunoprecipitation assay buffer (Beyotime, China). The protein extract was separated using 10% sodium dodecyl sulfate-polyacrylamide gel electrophoresis, transferred to a polyvinylidene difluoride (PVDF) membrane (Bio-Rad, Hercules, CA, USA), and blocked with skim milk powder for 1 h. The PVDF membrane was incubated with protein antibodies (1:1000, Abcam) at 4 °C overnight (collagen II, aggrecan, SOX9, MMP3, MMP13, ADAMTS4, and ADAMTS5). After washing with Tris-buffered saline with 0.1% Tween® 20, the cells were further incubated with the secondary antibody at room temperature (25 °C) for 2 h. Protein bands were observed using the LAS-4000 Science Imaging System (Fujifilm, Tokyo, Japan) and analyzed with ImageJ software (National Institutes of Health, Bethesda, MD, USA).

### Immunofluorescence

NPCs were cultured in 20 mm glass-bottom cell culture dishes (801001; Nest Biotechnology Co., Ltd., Shanghai, China). NPCs were fixed with 4% paraformaldehyde for 20 min. After washing with PBS, the cells were permeated with 0.3% Triton X-100 for 30 min and then blocked with 5% bovine serum albumin for 60 min. Cells were then incubated overnight at 4 °C with collagen II, aggrecan, MMP3, or ADAMTS4 antibodies (1:200; Abcam). The next day, the cells were washed with PBS and incubated with goat anti-rabbit IgG conjugated with the fluorescent dye Cy5 (1:100; Abcam). For nuclear staining, 4′,6-diamidino-2-phenylindole (Life Technologies, Carlsbad, CA, USA) was used. Immunofluorescence images were obtained using Nikon Eclipse TI and Zeiss LSM780 confocal microscopes and processed using Image-Pro Plus 6.0 (NIH, Bethesda, MD, USA).

### RNA FISH

The Cy3-labeled circ*SPG21* probe and 488-labeled locked nucleic acid miR-1197 probe were designed and synthesized by Haoke (Wuhan, China). A FISH kit (RiboBio, Guangzhou, China) was used to detect probe signals in human NPCs. Images were acquired using a Nikon A1Si laser scanning confocal microscope (Nikon Instruments Inc., Japan).

### Statistical analysis

Statistical analysis was performed using SPSS v22.0. The unpaired data between the two groups were tested using the *t*-test (using the 95% confidence interval for differences between groups). Differences between groups with values of *p* < 0.05 were considered significant.

## Results

### Comparison of normal and degenerative intervertebral discs

**Fig. 1:**
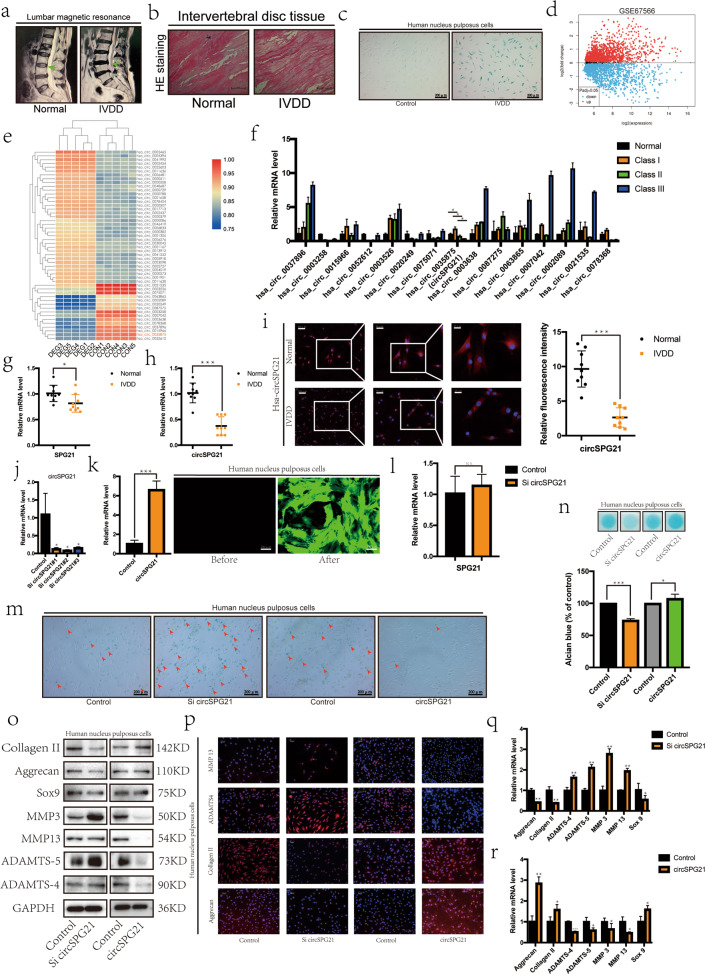
circ*SPG21* regulates NPC extracellular matrix (ECM) metabolism. **a** MRIs were compared between the normal and degenerative groups; *n* = 2 (two different donors). **b** HE staining was performed in the normal and degenerative group samples (scale bar, 100 μm); *n* = 2 (two different donors). c The degree of cell senescence was detected by β-galactosidase staining (scale bar, 200 μm); *n* = 2 (two different donors). **d** A volcano map was drawn according to the GEO database (GSE67566). **e** Heat map of all differentially expressed circular RNAs (circRNAs) in the degenerated and control disc tissues. **f** After screening 15 circRNAs, the relationship between circRNAs and the severity of degeneration was determined by real-time quantitative polymerase chain reaction (RT-qPCR), *n* = 4 (four different donors), *p < 0.05. **g** The expression of *SPG21*, as measured by RT-qPCR, in human degenerated tissue differed significantly from that in the control group; *n* = 6, *p < 0.05. Data are presented as the mean ± S.D., and the *p* values were determined by a two-tailed unpaired Student’s *t*-test. **h** The expression of circ*SPG21*, as measured by RT-qPCR, in human degenerated tissue was significantly lower than that in control tissue; *n* = 6, ***p < 0.001. Data are presented as the mean ± S.D., and the *p* values were determined by a two-tailed unpaired Student’s *t*-test. **i** Left, the expression of circ*SPG21*, as measured by FISH analysis, in human degenerated tissue was lower than that in control tissue. Representative images (scale bar, 25–100 μm) are displayed (two different donors). Right panel, the intensity of circ*SPG21* expression, as analyzed using fluorescence intensity, ***p < 0.001. Data are presented as the mean ± S.D., and the *p* values were determined by a two-tailed unpaired Student’s *t*-test. **j** Nucleus pulposus cells (NPCs) were transfected with circ*SPG21* siRNA# 1, 2, 3, or negative control siRNA (concentration 20 nm). The expression level of *circSPG21*, as determined using RT-qPCR, *p < 0.05. Data are presented as the mean ± S.D., and the *p* values were determined by a two-tailed unpaired Student’s *t*-test. **k** circ*SPG21* overexpression in NPCs after adenovirus infection with circ*SPG21*. Left, circ*SPG21* was highly expressed in NPCs. Right, after virus infection, green fluorescence was observed under a fluorescence microscope (scale bar, 200 μm); ***p < 0.001. Data are presented as the mean ± S.D., and the *p* values were determined by a two-tailed unpaired Student’s *t*-test. **l** Expression of *SPG21*, as detected by RT-qPCR, after transfection with si-circ*SPG21*. Data are presented as the mean ± S.D., and the *p* values were determined by a two-tailed unpaired Student’s *t*-test. **m** β-Galactosidase staining was performed to evaluate cell aging (scale bar, 200 μm). **n** Alcian staining was performed to evaluate the extracellular matrix; *p < 0.05, **p < 0.01. Data are presented as the mean ± S.D., and the *p* values were determined by a two-tailed unpaired Student’s *t*-test. **o** Expression of proteins (collagen II, aggrecan, SRY-box transcription factor 9 (SOX9), matrix metallopeptidase 3 (MMP3), MMP13, ADAM metallopeptidase with thrombospondin type 1 motif 4 (ADAMTS4), and ADAMTS5), as determined by western blotting. **p** Expression of collagen II, aggrecan, ADAMTS4, and MMP13, as determined by immunofluorescence (IF), compared according to the fluorescence intensity (Scale bar, 100 μm). Expression of genes after circ*SPG21* knockdown (**q**) or overexpression (**r**), as measured by RT-qPCR; *p < 0.05, **p < 0.01. Data are presented as the mean ± S.D., and the *p* values were determined by a two-tailed unpaired Student’s *t*-test.

In the clinical analysis, when a patient showed intervertebral disc degeneration, the high signal for the intervertebral disc in T2 MRI images disappeared, indicating water loss from the degenerative intervertebral disc (Fig. 1a). After obtaining consent from the patients, we collected the intervertebral discs of patients with spinal fracture due to trauma as the control group and those from patients with intervertebral disc degeneration as the degeneration group. After HE staining, the fibrous tissue in the degenerative group appeared loose compared with that in the control group, and the NPCs formed cell clusters (Fig. 1b). We extracted NPCs from the surgical specimens for β-galactosidase staining. Most cells in the degenerative group were green, indicating cell aging (Fig. 1c). Based on these results, when intervertebral disc degeneration occurs, a series of changes also occur in NP tissue and cells. The factor affecting the activity of NPCs could thus be a new target for treating disc degeneration.

### circ*SPG21* is expressed at low levels in degenerative tissues

In the study of RNAs, it was discovered that the interaction between circRNAs and miRNAs acts as a key regulator of gene expression. Therefore, an integrated microarray study (GSE67566) was performed on five control samples and five degeneration samples (Fig. 1d)^22^. In accordance with previous studies, circRNAs with significant differences between the control and degeneration groups (logFC < −1 or logFC > 1, *p* < 0.05) were selected to generate a heat map (Fig. 1e). circRNAs that exhibited significantly lower levels in the degenerative group might have a protective effect on disc degeneration; thus, 15 circRNAs with decreased expression in the heat map were selected. To verify the heat map results, patients with fractures were selected as the control group, and three patients with different degrees of lesions were selected according to the Pfirrmann classification^23^. These samples were then analyzed using real-time (RT) quantitative polymerase chain reaction (qPCR), and the expression level of hsa_circ_0035875 was found to decrease with increasing lesion severity (Fig. 1f; Supplementary Fig. 1a). This suggested a protective effect of hsa_circ_0035875, which is a loop formed by exons 2–3 of the *SPG21* gene (circ*SPG21*), on degeneration of the intervertebral disc.

Three representative from the spinal fracture samples group and three samples from the intervertebral disc degeneration group were selected for analysis. The expression levels of *SPG21* and circ*SPG21* were then measured in the samples using RT-qPCR. The results showed that the decrease in circ*SPG21* levels was greater than the decrease in *SPG21* levels in degenerative tissues, which excluded an effect of *SPG21* on circ*SPG21* (Fig. 1g, h). RNA fluorescence in situ hybridization (FISH) showed a decreased fluorescence intensity in the degenerated group (Fig. 1i). Therefore, we next explored the role and mechanism of circ*SPG21* in the intervertebral disc.

### circ*SPG21* regulates NPC extracellular matrix (ECM) metabolism

Small-interfering RNA was transfected into NPCs to inhibit the expression of circ*SPG21*^24^, and NPCs were also infected with adenovirus^25^ carrying the circ*SPG21* plasmid; circ*SPG21* expression was increased or decreased accordingly (sicirc*SPG21#1&2* were selected; Fig. 1j, k). When circ*SPG21* expression was suppressed, *SPG21* expression was not affected, and an influence of circ*SPG21* on *SPG21* was thus ruled out (Fig. 1l). The β-galactosidase staining results showed that circ*SPG21* effectively inhibited the senescence of NPCs (Fig. 1m). Circ*SPG21* also inhibited the degradation of the ECM based on Alcian blue staining (Fig. 1n). Additionally, western blotting revealed that the inhibition of circ*SPG21* expression promoted the expression of matrix metallopeptidase (MMP) 3, MMP13, ADAM metallopeptidase with thrombospondin type 1 (ADAMTS) motif 4, and ADAMTS5 and reduced the expression of collagen II, aggrecan, and SRY-box transcription factor 9 (SOX9). Increasing the expression of circ*SPG21* yielded the opposite results (Fig. 1o) based on immunofluorescence (IF) assays^26^ and RT-qPCR (Fig. 1p, q, r). These results indicated that circ*SPG21* inhibited catabolism and enhanced anabolism.

### Ring structure of circ*SPG21*

**Fig. 2:**
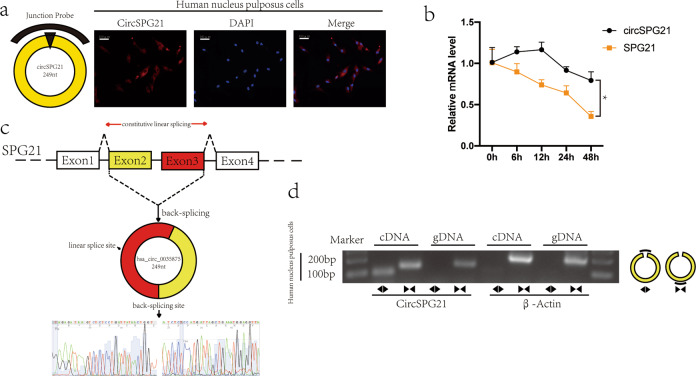
Ring structure of circSPG21. **a** Left, circ*SPG21* and probe diagram. Right, RNA-fluorescence in situ hybridization (FISH) showed that circ*SPG21* was mainly present in the cytoplasm (scale bar, 100 μm). The circular RNA probe was labeled with Cy-3. Nuclei were stained with 4′,6-diamidino-2-phenylindole (DAPI). **b** Nucleus pulposus cells were treated with 5 μg/mL actinomycin D, and the expression levels of circ*SPG21* and *SPG21* were detected by RT-qPCR, *p < 0.05. Data are presented as the mean ± S.D., and the *p* values were determined by a two-tailed unpaired Student’s *t*-test. **c** Exons 2–3 of *SPG21* form circ*SPG21*. Sanger sequencing confirmed the presence of circ*SPG21*. **d** Left: circ*SPG21* was amplified by divergent and convergent primers in cDNA and genomic DNA and separated by horizontal electrophoresis. β-actin served as a negative control. Right: a diagram of the divergent and convergent primers.

RNA FISH^14^ revealed that circSPG21 mainly localized to the cytoplasm (Fig. 2a). After actinomycin D treatment (an inhibitor of transcription), the expression of circ*SPG21* and *SPG21* was detected at various time points (0, 6, 12, 24, 48 h). The half-life of circ*SPG21* was more than 48 h, indicating that circ*SPG21* was stable in NPCs (Fig. 2b). circRNA forms a ring structure through a head-to-tail connection; this structure differs from that of ordinary RNA in that there is no 5ʹ hat or 3ʹ poly(A) structure. To clarify this structure, the sequence at the circ*SPG21* junction was examined using Sanger sequencing, and it was found that circ*SPG21* was formed by the splicing of exons 2–3 of *SPG21* (Fig. 2c). To exclude the possibility of trans-splicing^27^ (two different pre-mRNA exons were linked together, which has been observed in various species), a convergent primer was designed to amplify *SPG21* mRNA, and a divergent primer was designed to amplify circ*SPG21* from both cDNA and genomic DNA (gDNA). Bands were amplified from cDNA with both primers; however, only the *SPG21* band was amplified from gDNA (Fig. 2d). This evidence indicated that circ*SPG21* formed a stable ring structure via the end-to-end connection of *SPG21* mRNA and was widely present in the cytoplasm.

### ceRNA network construction

**Fig. 3:**
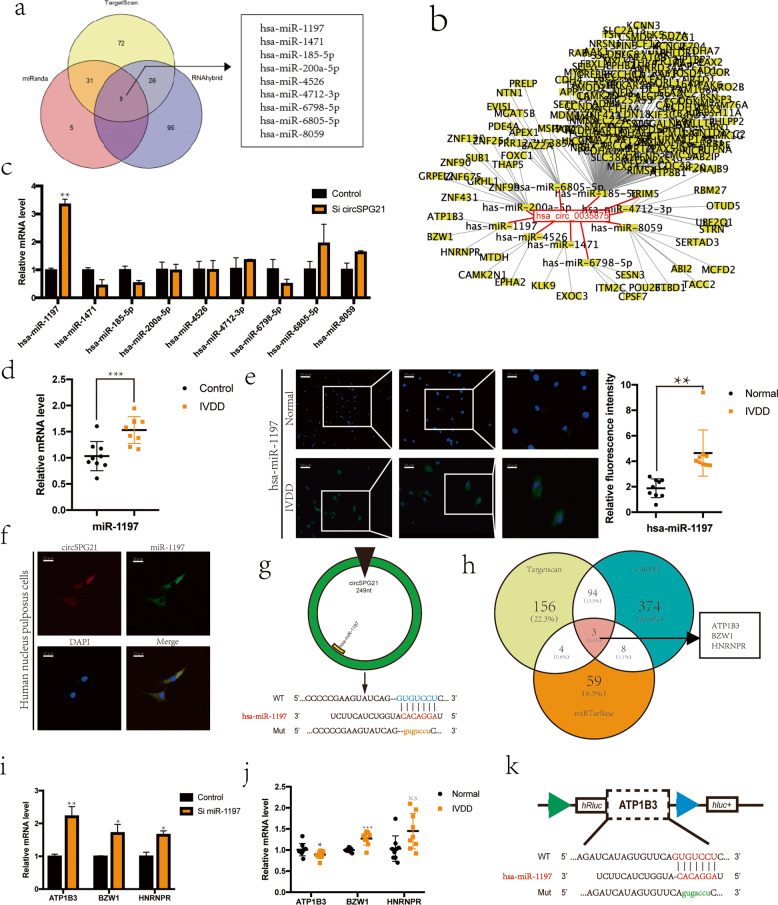
ceRNA network construction. **a** circ*SPG21*-target microRNA was predicted based on the intersection of the TargetScan, miRanda, and RNAhybrid software data. **b** A ceRNA network diagram was drawn using Cytoscape. **c** Expression of microRNAs after circ*SPG21* knockdown, as detected by real-time quantitative polymerase chain reaction (RT-qPCR), **p < 0.01. Data are presented as the mean ± S.D., and the *p* values were determined by a two-tailed unpaired Student’s *t*-test. **d** miR-1197 expression increased in degenerated tissues, as measured by RT-qPCR; *n* = 6, ***p < 0.001. Data are presented as the mean ± S.D., and the *p* values were determined by a two-tailed unpaired Student’s *t*-test. **e** Left, the expression of miR-1197, as measured by fluorescence in situ hybridization (FISH), in degenerated tissues was higher than that in control tissues (scale bar, 100 μm). Right panel, the fluorescence intensity of miR-1197 was stronger in degenerated tissue (two different donors), **p < 0.01. Data are presented as the mean ± S.D., and the *p* values were determined by a two-tailed unpaired Student’s *t*-test. The miR-1197 probe was labeled with Alexa Fluor 488. **f** circ*SPG21* and miR-1197 were localized in the cell, and both were seen in the cytoplasm (scale bar, 25 μm). **g** The sites where circ*SPG21* and miR-1197 bind to each other were identified using CircInteractome. **h** The target genes of miR-1197 were predicted based on the intersection of TargetScan, miRDB, and miRTarBase results. **i** Expression of target genes after miR-1197 knockdown, as detected by RT-qPCR; *p < 0.05, **p < 0.01. Data are presented as the mean ± S.D., and the *p* values were determined by a two-tailed unpaired Student’s *t*-test. **j** The expression of *ATP1B3, BZW1*, and *HNRNPR* was detected in degenerated tissues using RT-qPCR; *p < 0.05, ***p < 0.001. Data are presented as the mean ± S.D., and the *p* values were determined by a two-tailed unpaired Student’s *t*-test. **k** The binding site of miR-1197 and *ATP1B3* predicted by TargetScan.

circRNA exists widely in eukaryotic cells and acts as an miRNA sponge. Using online databases (TargetScan: www.targetscan.org/; miRanda: www.miranda.org/; and RNAhybrid: RNA hybridization software), nine miRNAs that could stably bind circ*SPG21* were identified (Fig. 3a). The mechanistic circRNA–microRNA–target gene network was constructed using an online database (Fig. 3b). To verify the relationship between miRNA and circ*SPG21*, the expression of circ*SPG21* was inhibited in NPCs, and it was found that the expression of miR-1197 significantly increased (Fig. 3c; Supplementary Fig. 1b)^28^. Therefore, it was hypothesized that miR-1197 might be the downstream target of circ*SPG21* and could play a role in disc degeneration. The expression of miR-1197 was increased in degenerative tissues, as confirmed by both RT-qPCR (Fig. 3d) and FISH (Fig. 3e) assays. FISH analyses revealed that circ*SPG21* and miR-1197 colocalized in the cytoplasm, and there was an intersection in the cell space (Fig. 3f). The targets of the interaction between circ*SPG21* and miR-1197 were predicted using an online database (Fig. 3g). Therefore, miR-1197 was selected for further analysis.

Target genes of miR-1197 were predicted using three online databases (TargetScan, miRDB, and miRTarBase), and the genes common to the three prediction results were selected as the three most suitable target genes (Fig. 3h). The expression of miR-1197 was inhibited in NPCs, and it was found that among the three target genes, *ATP1B3* expression significantly increased; thus, it was hypothesized that *ATP1B3* might be the target gene of miR-1197 (Fig. 3i). Furthermore, the expression of *ATP1B3* was decreased in degenerated tissue of the intervertebral disc, and *ATP1B3* was therefore selected to explore its function and mechanism (Fig. 3j). The target sequence of miR1197 with respect to *ATP1B3* was predicted (Fig. 3k).

### MiR-1197 and *ATP1B3* affect the activity of NPCs

**Fig. 4:**
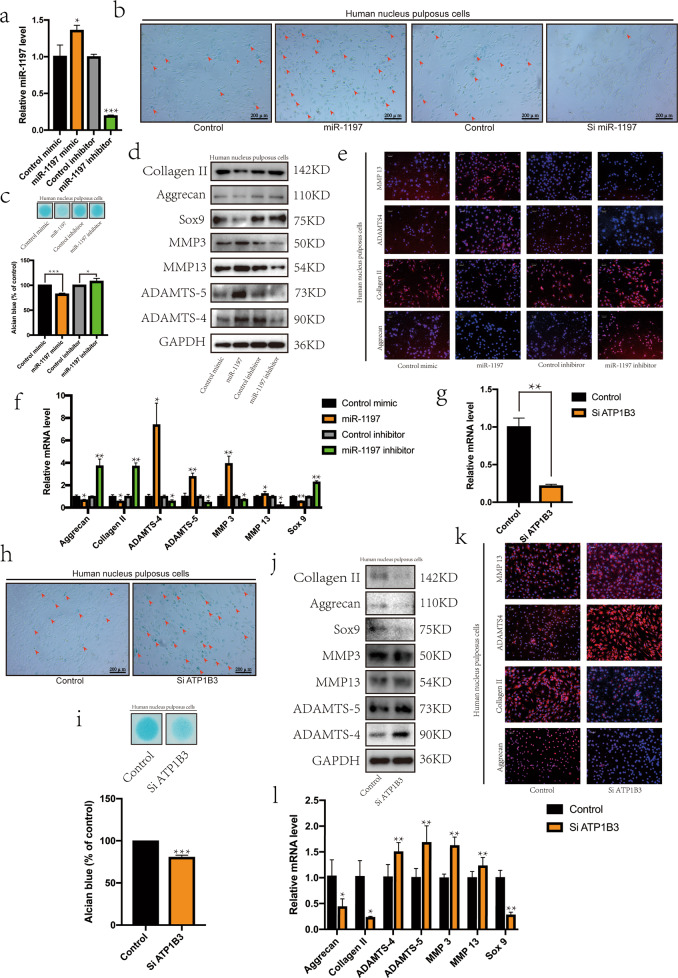
MiR-1197 and *ATP1B3* affect the activity of NPCs. **a** The efficiency of transfection with miR-1197 mimics and inhibitors, as determined by real-time quantitative polymerase chain reaction (RT-qPCR); *p < 0.05, ***p < 0.001. Data are presented as the mean ± S.D., and the *p* values were determined by a two-tailed unpaired Student’s *t*-test. **b** β-Galactosidase staining was performed to evaluate cell aging (scale bar, 200 μm). **c** Alcian staining was performed to evaluate the extracellular matrix; *p < 0.05, **p < 0.01. Data are presented as the mean ± S.D., and the *p* values were determined by a two-tailed unpaired Student’s t-test. **d** After the expression of miR-1197 was knocked down or miR-1197 was overexpressed, the expression of related proteins (collagen II, aggrecan, SRY-box transcription factor 9 (SOX9), matrix metallopeptidase 3 (MMP3), MMP13, ADAM metallopeptidase with thrombospondin type 1 motif 4 (ADAMTS4), and ADAMTS5) was detected by western blotting. **e** Expression of collagen II, aggrecan, ADAMTS4, and MMP13 was measured using immunofluorescence (IF) comparing the fluorescence intensity (scale bar, 100 μm). **f** Expression of related genes, as detected by RT-qPCR; *p < 0.05, **p < 0.01. Data are presented as the mean ± S.D., and the *p* values were determined by a two-tailed unpaired Student’s *t*-test. **g** The efficiency of siRNA targeting *ATP1B3* was tested using RT-qPCR; **p < 0.01. Data are presented as the mean ± S.D., and the *p* values were determined by a two-tailed unpaired Student’s *t*-test. **h** β-Galactosidase staining was performed to evaluate cell aging (scale bar, 200 μm). **i** Alcian staining was performed to evaluate the extracellular matrix. *p < 0.05, **p < 0.01. Data are presented as the mean ± S.D., and the *p* values were determined by a two-tailed unpaired Student’s *t*-test. **j** Expression of proteins (collagen II, aggrecan, SRY-box transcription factor 9 (SOX9), matrix metallopeptidase 3 (MMP3), MMP13, ADAM metallopeptidase with thrombospondin type 1 motif 4 (ADAMTS4), and ADAMTS5) after knocking down *ATP1B3*, as determined by western blotting. **k** Fluorescence intensity of proteins (collagen II, aggrecan, ADAMTS4, and MMP13) after the knockdown of *ATP1B3*, as determined by immunofluorescence (scale bar, 100 μm). **l** Expression of genes after the knockdown of *ATP1B3*, as detected by RT-qPCR; *p < 0.05, **p < 0.01. Data are presented as the mean ± S.D., and the *p* values were determined by a two-tailed unpaired Student’s *t*-test.

To study the role of miR-1197, its expression was downregulated or upregulated in NPCs (Fig. 4a). miR-1197 accelerated the senescence of NPCs (Fig. 4b) and decomposition of the ECM (Fig. 4c). From the western blot results, it was observed that the expression of collagen II, aggrecan, and SOX9 decreased, but the expression of MMP3, MMP13, ADAMTS4, and ADAMTS5 increased, when miR-1197 was overexpressed. The opposite results were obtained when miR-1197 was knocked down (Fig. 4d). IF and RT-qPCR both showed similar results (Fig. 4e, f). Therefore, circ*SPG21* can affect ECM metabolism by NPCs through miR-1197.

Next, the efficiency of *ATP1B3* siRNA was tested (Fig. 4g). When *ATP1B3* expression was decreased, NPCs displayed accelerated senescence (Fig. 4h) and ECM decomposition (Fig. 4i), indicating that *ATP1B3* exerts a protective effect on NPCs. In addition, the expression of collagen II, aggrecan, and SOX9 decreased, whereas the expression of MMP3, MMP13, ADAMTS4, and ADAMTS5 increased, as observed by western blotting (Fig. 4j) and IF (Fig. 4k). Similar results were observed using RT-qPCR (Fig. 4l). These results indicated that *ATP1B3* had a protective effect on NPCs.

### Verification of the circSPG21/miR-1197/ATP1B3 axis in NPCs

**Fig. 5:**
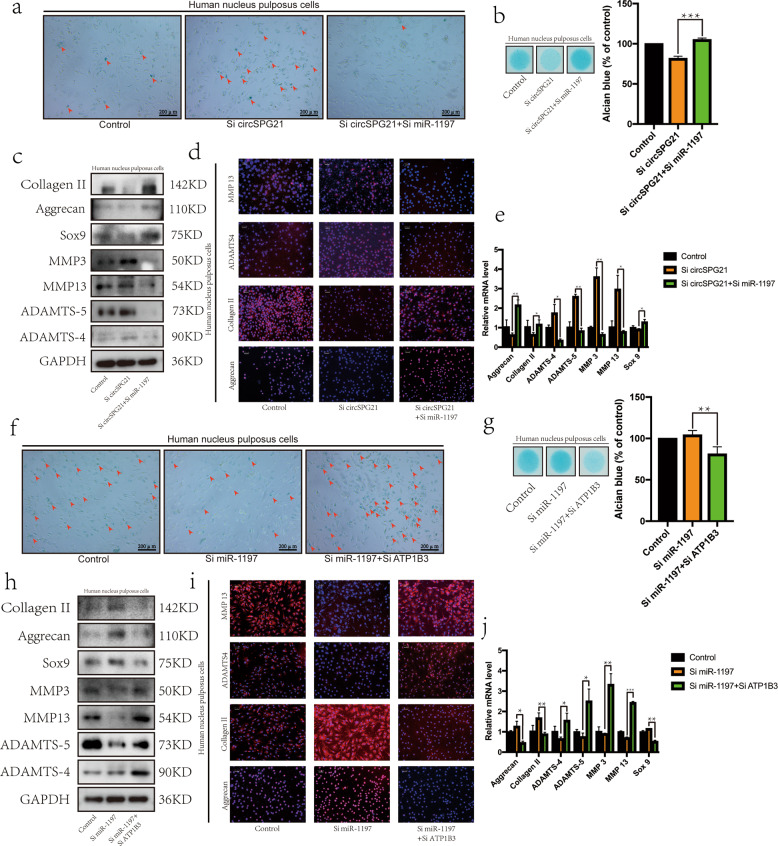
Verification of the circSPG21/miR-1197/ATP1B3 axis in NPCs. **a** β-galactosidase staining was performed to evaluate cell aging (scale bar, 200 μm). **b** Alcian staining was performed to evaluate the extracellular matrix; *p < 0.05, **p < 0.01. Data are presented as the mean ± S.D., and the *p* values were determined by a two-tailed unpaired Student’s *t*-test. **c** Changes in the expression of proteins (collagen II, aggrecan, SRY-box transcription factor 9 (SOX9), matrix metallopeptidase 3 (MMP3), MMP13, ADAM metallopeptidase with thrombospondin type 1 motif 4 (ADAMTS4), and ADAMTS5) when circ*SPG21* was knocked down, either alone or in combination with miR-1197. **d** Fluorescence intensity of collagen II, aggrecan, ADAMTS4, and MMP13 based on immunofluorescence (IF; scale bar, 100 μm). **e** Expression of genes (collagen II, aggrecan SOX9, MMP3, MMP13, ADAMTS4, and ADAMTS5), as detected by real-time quantitative polymerase chain reaction (RT-qPCR); *p < 0.05, **p < 0.01. Data are presented as the mean ± S.D., and the *p* values were determined by a two-tailed unpaired Student’s *t*-test. **f** β-Galactosidase staining was performed to evaluate cell aging (scale bar, 200 μm). **g** Alcian staining was performed to evaluate the extracellular matrix. *p < 0.05, **p < 0.01. Data are presented as the mean ± S.D., and the *p* values were determined by a two-tailed unpaired Student’s *t*-test. **h** Expression of proteins (collagen II, aggrecan SOX9, MMP3, MMP13, ADAMTS4, and ADAMTS5) when miR-1197 was knocked down, either alone or in combination with ATP1B3, as determined by western blotting. **i** Fluorescence intensity of proteins (collagen II, aggrecan, ADAMTS4, and MMP13) when miR-1197 was knocked down, either alone or in combination with *ATP1B3*, as determined by IF (scale bar, 100 μm). **j** Expression of genes when miR-1197 was knocked down, either alone or in combination with *ATP1B3*, as detected by RT-qPCR; *p < 0.05, **p < 0.01, ***p < 0.001. Data are presented as the mean ± S.D., and the *p* values were determined by a two-tailed unpaired Student’s *t*-test.

To determine whether the function of circ*SPG21* affects IVDD via miR-1197, a rescue experiment was designed in which the results of knocking down circ*SPG21* expression alone were compared with those of knocking down circ*SPG21* and miR-1197 expression at the same time. The knockdown of miR-1197 by decreasing the expression of circ*SPG21* successfully rescued the aging trend of NPCs (Fig. 5a) and inhibited the decomposition of the ECM (Fig. 5b). Similarly, when cells were transfected with circ*SPG21* alone, matrix-synthesizing proteins (collagen II, aggrecan, and SOX9) were inhibited, and the expression of degrading enzymes (MMP3, MMP13, ADAMTS4, and ADAMTS5) was promoted. However, these results were reversed when circ*SPG21* and miR-1197 were cotransfected (Fig. 5c). The IF and RT-qPCR results also showed that the cotransfection of circ*SPG21* and miR-1197 salvaged gene and protein expression (Fig. 5d, e). In conclusion, as a downstream molecule of circ*SPG21*, miR-1197 aggravates NP degeneration.

Next, the relationship between miR-1197 and *ATP1B3* was explored using a rescue experiment. The knockdown of *ATP1B3* (based on the low expression of miR-1197) accelerated the aging trend of NPCs (Fig. 5f) and the decomposition of the ECM (Fig. 5g). Similarly, when miR-1197 expression decreased, the expression of collagen II, aggrecan, and SOX9 increased, whereas the expression of MMP3, MMP13, ADAMTS4, and ADAMTS5 decreased at both the gene and protein levels. Cotransfection with si-miR-1197 and si-*ATP1B3* yielded the opposite expression trend (Fig. 5h–j). These results indicated that *ATP1B3* is the downstream effector of miR-1197.

### Injection of circ*SPG21* alleviates IVDD in a mouse model

**Fig. 6:**
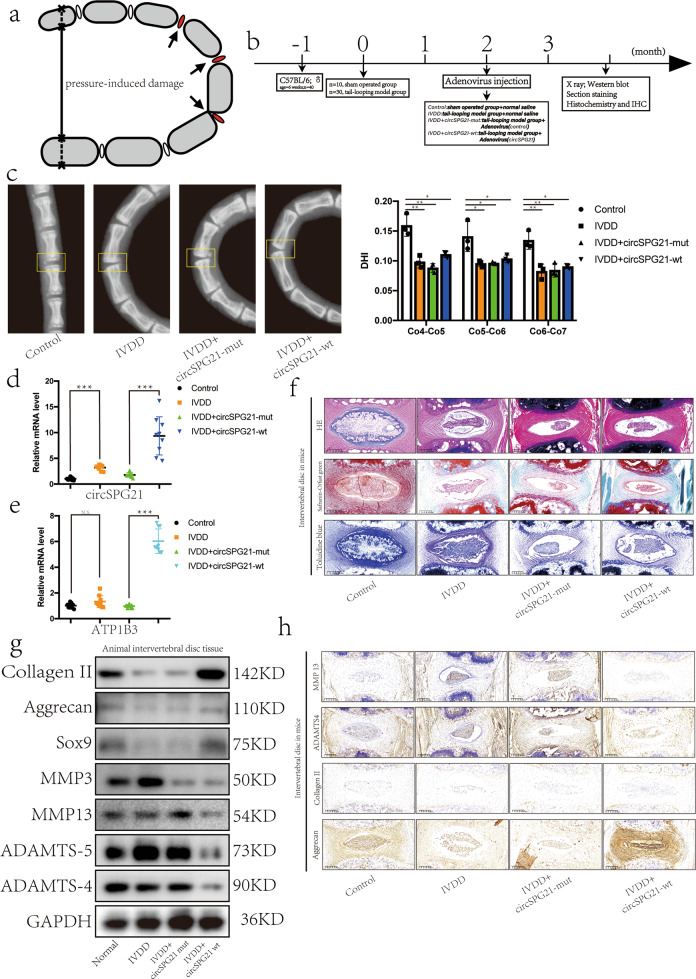
Injection of circ*SPG21* alleviates IVDD in a mouse model. **a** Schematic diagram showing the composition and principle of the tail-looping model. **b** Diagram showing the time sequence of in vivo experiments. **c** Left, the tails of mice, as observed by X-ray. Right panel, disc height index (DHI) of coccyx4–coccyx5, coccyx5–coccyx6, and coccyx7–coccyx8; *n* = 12, *p < 0.05, **p < 0.01. Data are presented as the mean ± S.D., and the *p* values were determined by a two-tailed unpaired Student’s *t*-test. **d** Expression of circ*SPG21* in the intervertebral discs of mice after the injection of adenovirus or saline, as detected by real-time quantitative polymerase chain reaction (RT-qPCR); *n* = 12, ***p < 0.001. Data are presented as the mean ± S.D., and the *p* values were determined by a two-tailed unpaired Student’s *t*-test. **e** Expression of *ATP1B3* in the intervertebral disc of mice, as detected by RT-qPCR; *n* = 12, ***p < 0.001. Data are presented as the mean ± S.D., and the *p* values were determined by a two-tailed unpaired Student’s *t*-test. **f** Tissue sections of mice stained with hematoxylin and eosin (HE), safranin-O/fast green, and toluidine blue to show the degree of intervertebral disc degeneration (scale bar, 200 μm). **g** Expression of collagen II, aggrecan, SRY-box transcription factor 9 (SOX9), matrix metallopeptidase 3 (MMP3), MMP13, ADAM metallopeptidase with thrombospondin type 1 motif 4 (ADAMTS4), and ADAMTS5 in intervertebral disc tissue, as determined by western blotting. **h** Expression of proteins (collagen II, aggrecan, ADAMTS4, and MMP13), as determined by immunohistochemical staining (scale bar, 200 μm).

As shown in Fig. 6a, the tail of a mouse was fixed to form a ring by penetrating the mouse tail bone with a thin wire; thus, the pressure on the intervertebral disc tissue in the tails of the mice was increased. Under pressure, intervertebral disc tissue degenerates. Six-week-old male mice were selected, and their tails were fixed for two months to form a tail-looping model^18^. Then, the experimental group was intraperitoneally injected with adenovirus carrying the circ*SPG21* plasmid, and the control group was injected with normal saline once per week for eight weeks (Fig. 6b). The tails were subsequently removed, and radiographs were taken. The caudate bone in the tail-looping model mice was morbidly angulated, and the disc height index was decreased, indicating compression of the tail disc (Fig. 6c). circ*SPG21* and *ATP1B3* expression was increased in mice injected with the circ*SPG21*-wt plasmid, as measured by RT-qPCR (Fig. 6d, e). The tails of the mice were then paraffin sectioned and stained with HE, safranin-O/fast green, and toluidine blue. Relative to normal tissue, the degenerative disc tissue showed a loss of NP tissue (Fig. 6f). Total protein was next extracted from the tail intervertebral disc tissue for western blotting (Fig. 6g). The results showed that anabolic activity decreased and catabolism increased in degenerative tissues. However, increased circ*SPG21* expression changed this trend and protected the NP; this result was confirmed by immunohistochemistry (Fig. 6h).

## Discussion

The development of new treatment alternatives for IVDD has been stagnating; thus, it is imperative to explore its pathogenesis to identify new targets. circRNAs are widely expressed in mammalian cells, show tissue specificity, have a conserved and stable structure, and offer great potential for the development of new treatments. Several studies have shown that circRNAs are associated with human diseases. However, little is known about their mechanism in the occurrence and development of IVDD, which suggests that this should be explored^8^.

**Fig. 7:**
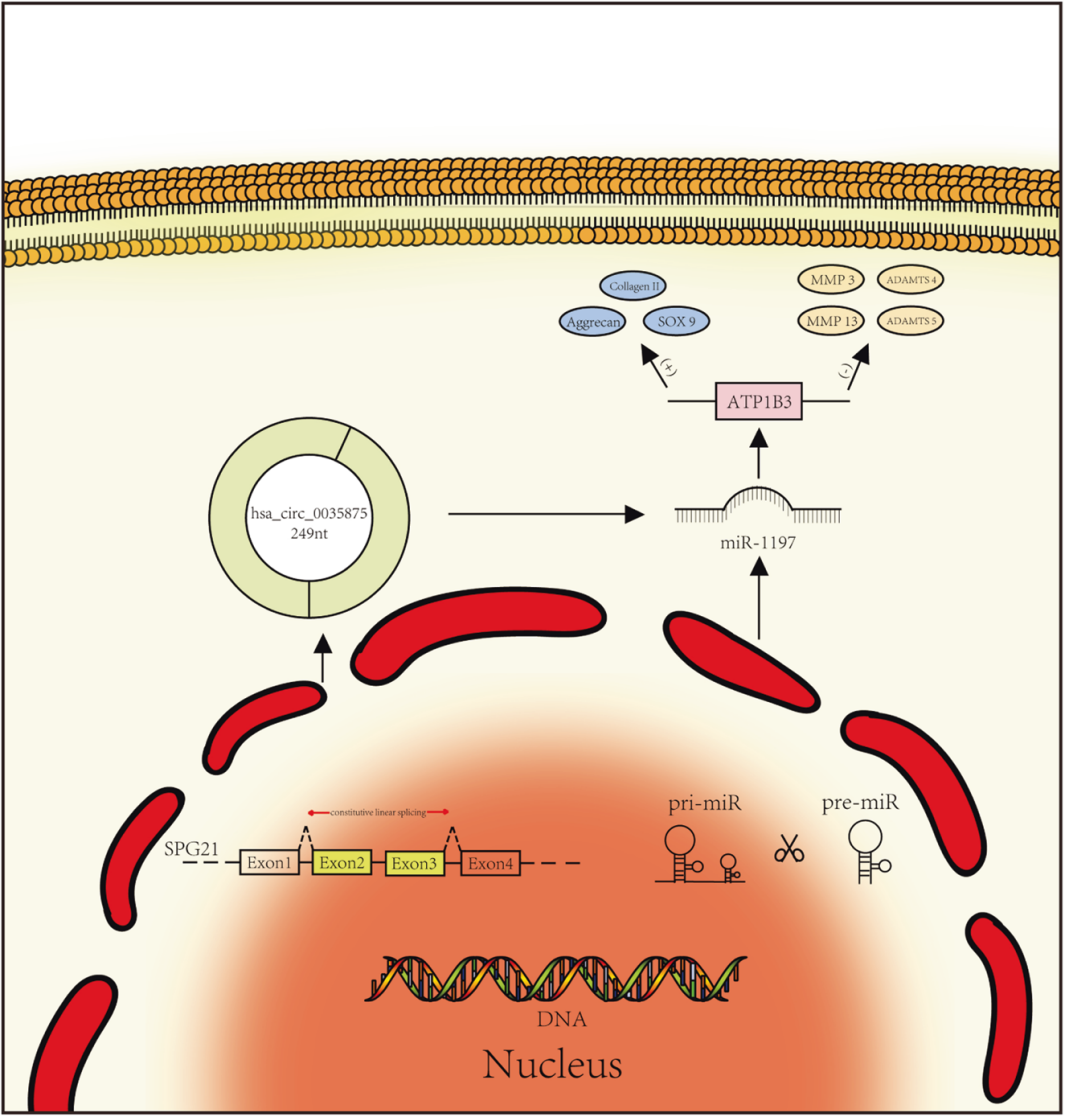
Schematic diagram of circSPG21 mechanism. A schematic diagram of the mechanism underlying the effect of circular RNA.

In this study, it was first determined that circ*SPG21* is the key circRNA involved in IVDD. circ*SPG21* was determined to be involved in the synthesis and degradation of IVDD-related proteins. The potential impact of circ*SPG21* on the regulatory function of miRNAs in NPCs was then studied. circ*SPG21* significantly reduced activity and function by capturing miR-1197. Therefore, it was hypothesized that miR-1197 was a downstream target molecule of circ*SPG21* that promoted anabolism and inhibited catabolism. Furthermore, the overexpression of *ATP1B3* induced by miR-1197 gene knockout exerted a protective effect on IVDD. Therefore, a prospective strategy of targeting the circ*SPG21*/miR-1197/*ATP1B3* axis for IVDD treatment was proposed (Fig. 7). Previous studies have shown that intervertebral disc degeneration is closely related to inflammation. However, we identified downstream target genes that suggested that intervertebral disc degeneration might be related to the intracellular ion concentration. *ATP1B3* encodes an Na^+^/K^+^ ion channel protein in the cell membrane. In degenerative tissues, decreased *ATP1B3* expression prevents the exchange of Na^+^/K^+^ ions. We hypothesize that Na^+^/K^+^ ions accumulate in cells in degenerated tissues, intracellular osmotic pressure rises, and water passively enters cells, causing a deformed cell morphology. However, the relationship between the changes in ion concentrations and IVDD warrants further study.

To date, research on the ceRNA network has shown that the interacting factors in the network are closely related to the occurrence and development of human diseases. circWHSC1 is considerably more highly expressed in endometrial carcinoma than in normal tissues. Through bioinformatics prediction and experimental verification, the circWHSC1/miR-646/NPM1 axis was found to promote the development of endometrial cancer^29^. Through the microarray analysis of metastatic and nonmetastatic human hepatocellular carcinoma tissues, the lncRNA CDKN2BAS was found to be significantly upregulated in metastatic tumors. With expanding research, CDKN2BAS was determined to regulate miR-153-5p and act as a ceRNA, which could increase the expression of *ARHGAP18*, the target gene of miR-153-5p^30^. The ceRNA network is a platform that modulates protein-encoding mRNA and nonencoding RNA and might represent a wide range of posttranscriptional regulatory events related to gene expression involved in physiology and pathology. With accumulating research, ceRNA network interactions could be exploited in RNA-directed therapy.

However, there are limitations to this study. Age differences between patients with and without IVDD might have led to some bias. Moreover, because of the clinicopathological characteristics of IVDD, the detection of NP tissue is an inevitable confounding factor. Therefore, animal model experiments were conducted with the hope that this approach would help eliminate the influence of age and individual factors. In addition to its ceRNA function, there could be other potential mechanisms through which circ*SPG21* regulates IVDD, such as by interacting with RNA-binding proteins^31^. Furthermore, the expression level of circ*SPG21* decreased during the degeneration of NPCs. We speculate that there is a complex regulatory mechanism upstream of circ*SPG21*. The specific mechanism of circ*SPG21* thus still needs to be further explored. In conclusion, circ*SPG21* reduces the apoptosis of NPCs induced by inflammatory cytokines and the imbalance of ECM anabolism and catabolism through miR-1197/*ATP1B3*. Thus, targeting circ*SPG21* may provide an effective treatment strategy for IVDD.

## Supplementary information

Supplementary material

## Data Availability

All data included in this study are available upon request by contacting the corresponding author.
