# Supplementary material for: circSPG21 protects against intervertebral disc disease by targeting miR-1197/ATP1B3
Source: Exp Mol Med. 2021 Oct 6;53(10):1547–58. doi: 10.1038/s12276-021-00674-z (PMC8568895; doi:10.1038/s12276-021-00674-z)
Supplement: Supplementary file 1 — Supplementary material [file 12276_2021_674_MOESM1_ESM.pdf]

Supplementary Materials

circSPG21 protects against intervertebral disc disease by targeting miR-1197/_ATP1B3_

Yizhen Huang, Zhenlei Zhang, Jianle Wang, Shuying Shen, Teng Yao, Yining Xu, Zizheng Chen, Bin Fang, and Jianjun Ma

Supplementary Table 1:
Patient information summary
Control

Number	Sex	Age (year)	Height (cm)	Weight (kg)	BMI
55xxx06	Male	33	170	78.5	27.16263
31xxx57	Female	36	160	51	19.92188
55xxx18	Female	51	152	46	19.90997

IVDD

Number	Sex	Age (year)	Height (cm)	Weight (kg)	BMI
50xxx96	Female	24	156	70	28.76397
30xxx98	Female	32	171	64	21.88708
56xxx30	Male	34	169	58.8	20.58751
55xxx50	Female	42	154	46	19.39619
55xxx97	Male	43	185	80	23.37473
55xxx95	Female	46	160	65.6	25.625
56xxx06	Female	47	158	67.5	27.03894
46xxx90	Female	49	160	52.5	20.50781
55xxx03	Female	50	159	75	29.66655
57xxx35	Male	52	168	66.85	23.68552
56xxx11	Male	54	172	68	22.9854
53xxx50	Female	54	160	58.5	22.85156
24xxx09	Female	54	156	61	25.06575
80xxx03	Male	54	155	51	21.22789
54xxx99	Female	55	160	60	23.4375
43xxx76	Male	56	170	73.8	25.53633
56xxx01	Male	56	171	80	27.35885
29xxx98	Female	56	158	59	23.63403
56xxx56	Male	56	171	80	27.35885
56xxx20	Male	56	167	65	23.30668
28xxx94	Female	57	160	60	23.4375
18xxx67	Female	57	157	56	22.71897
81xxx38	Female	60	159	75	29.66655

56xxx00	Male	60	170	62	21.45329
56xxx62	Female	60	146	47.6	22.33064
55xxx15	Female	62	155	52	21.64412
56xxx07	Female	62	160	74	28.90625
55xxx78	Female	62	150	55.6	24.71111
56xxx16	Male	63	175	90	29.38776
36xxx62	Male	63	169	87	30.46112
56xxx90	Female	63	158	65	26.03749
56xxx35	Male	63	168	72	25.5102
56xxx88	Male	64	175	68	22.20408
56xxx43	Male	65	160	65.5	25.58594
54xxx86	Male	65	176	62	20.0155
51xxx33	Female	66	154	53	22.34778
20xxx56	Male	67	170	85	29.41176
54xxx24	Male	68	170	54	18.68512
32xxx29	Male	69	173	68	22.72044
55xxx67	Male	70	175	65	21.22449
55xxx21	Female	70	158	68	27.23922
44xxx05	Female	70	152	41.5	17.96226
36xxx09	Female	72	155	66.5	27.6795
56xxx65	Male	73	160	75	29.29688
56xxx37	Male	73	168	69	24.44728
23xxx59	Male	73	170	65.5	22.66436
80xxx68	Female	75	158	56	22.4323
19xxx68	Male	75	172	82	27.71769
55xxx44	Male	82	168	68	24.09297

Supplementary Table 2:
Primer sequence summary

circ_0035875 F	GGGTTATCGCTGATTAACCTCCA
circ_0035875 R	GGGGAACTGTACCTCTAAACCA
hsa_circ_0037896 F	TCCTGGGAAAGCTGGAATCAG
hsa_circ_0037896 R	AATTGGCAGCCTGATTGGTCC
hsa_circ_0003258 F	GCACAAACAGTTCATGTGCCA
hsa_circ_0003258 R	AAGGAGTGCACCTTGAGTGA
hsa_circ_0015966 F	TCCGAAGCATCTTAGCCGATG
hsa_circ_0015966 R	TCCTGATCACCCACCAAGTG
hsa_circ_0052612 F	AACTCAGGCATGCTTCTGAC
hsa_circ_0052612 R	GGGGTAGAGACAAACATCATTAAG
hsa_circ_0003526 F	GCAATTCCTTCAGTGACACCT
hsa_circ_0003526 R	GGGAACTGTACCTCTAAACCAGT
hsa_circ_0020249 F	TACACCAGGATCCACCCGAT
hsa_circ_0020249 R	TCTTCGCCATCCTCGGTCTA
hsa_circ_0075071 F	TGTTCCCTTTGCTGCCGTAG
hsa_circ_0075071 R	TGCTTTGATCCCATCGAGGTT
hsa_circ_0003638 F	TGAGGACAGCTTGTGAAGGC
hsa_circ_0003638 R	TTCACAAGGAGATTCCCTGGC
hsa_circ_0087275 F	GCTCCTTGTACAACACGCCT
hsa_circ_0087275 R	AAACTCCACACCATGCACCG
hsa_circ_0063865 F	CCCACCACTGCAAGATAACCA
hsa_circ_0063865 R	GCAGGTGCTCCATCAATGC
hsa_circ_0007042 F	GCGTCCTGGTCACCTTCAGA
hsa_circ_0007042 R	ATGGCTCAGTCCCAGGTAGT
hsa_circ_0002089 F	AGGAGCAGCATGGCATTTCA
hsa_circ_0002089 R	TCAGAAACTGTCTGTGCCACC
hsa_circ_0021535 F	CTGCCGAACAACCAAAGCAA
hsa_circ_0021535 R	AAGTCCATGAACGACCACCC
hsa_circ_0078368 F	ACTACTCTCTGGGTTGGGCA
hsa_circ_0078368 R	TCGCTTTCGAAATGGGTGGT
SPG21 F	GGTCGCTCTATGACGCGG
SPG21 R	CGATAACCCGGTAACCCCAT
h-MMP3-F	CCTACAAGGAGGCAGGCAAG
h-MMP3-R	CCCGTCACCTCCAATCCAAG
h-MMP13-F	TCGGCCACTCCTTAGGTCTT
h-MMP13-R	AAGTGGCTTTTGCCGGTGTA
h-COL2A1-F	CCAGATGACCTTCCTACGCC
h-COL2A1-R	TTCAGGGCAGTGTACGTGAAC
h-ADAMTS4 F	AACGTCAAGGCTCCTCTTGG
h-ADAMTS4 R	TGACAGGATTGCGGATGCTT

h-ADAMTS5 F	CCGGAGCCACTGCTTCTATC
h-ADAMTS5 R	ACCCCCACAGAGGTCAAAGA
h-Aggrecan	GGGACCTGCAAGGAGACAGAG
h-Aggrecan	TCAATCTCACACAGGTCCCCTTC
h-β-actin-F	AGAGCTACGAGCTGCCTGAC
h-β-actin-R	AGCACTGTGTTGGCGTACAG
h-SOX9 F	GCTCTGGAGACTTCTGAACGA
h-SOX9 R	CCGTTCTTCACCGACTTCCT

Supplementary Figure 1:

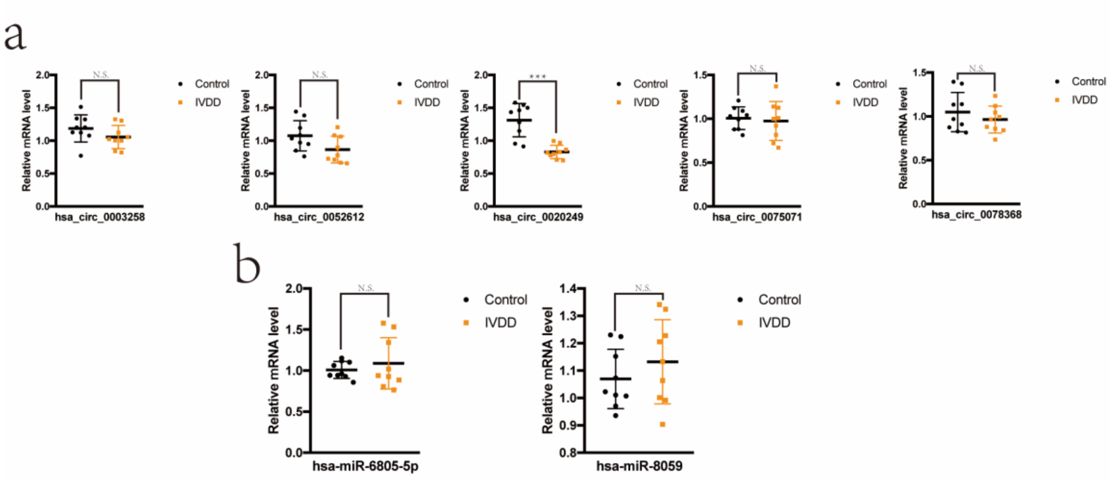

(a) The expression of hsa_circ_0003258, hsa_circ_0052612, hsa_circ_0020249, hsa_circ_0075071, hsa_circ_0078368, as measured by RT-qPCR, in human degenerated tissue significantly differed from that in the control group; n=6, ***$p < 0.001$. Data are presented as the mean ± S.D., and the *p*-values were determined by a two-tailed unpaired Student's t-test. (b) The expression of hsa-miR-6805-5p, hsa-miR-8059, as measured by RT-qPCR, in human degenerated tissue was significantly lower than that in control tissue; n=6. Data are presented as the mean ± S.D., and the *p*-values were determined by a two-tailed unpaired Student's t-test.
